# Differential Modulation of the Voltage-Gated Na^+^ Channel 1.6 by Peptides Derived From Fibroblast Growth Factor 14

**DOI:** 10.3389/fmolb.2021.742903

**Published:** 2021-09-07

**Authors:** Aditya K. Singh, Nolan M. Dvorak, Cynthia M. Tapia, Angela Mosebarger, Syed R. Ali, Zaniqua Bullock, Haiying Chen, Jia Zhou, Fernanda Laezza

**Affiliations:** ^1^ Department of Pharmacology and Toxicology, Galveston, TX, United States; ^2^ Pharmacology and Toxicology Graduate Program, Galveston, TX, United States; ^3^ Presidential Scholarship Program, University of Texas Medical Branch, Galveston, TX, United States

**Keywords:** voltage-gated sodium channels, protein-protein interactions, intracellular fibroblast growth factors, split-luciferase complementation assays, patch-clamp electrophysiology

## Abstract

The voltage-gated Na^+^ (Nav) channel is a primary molecular determinant of the initiation and propagation of the action potential. Despite the central role of the pore-forming α subunit in conferring this functionality, protein:protein interactions (PPI) between the α subunit and auxiliary proteins are necessary for the full physiological activity of Nav channels. In the central nervous system (CNS), one such PPI occurs between the C-terminal domain of the Nav1.6 channel and fibroblast growth factor 14 (FGF14). Given the primacy of this PPI in regulating the excitability of neurons in clinically relevant brain regions, peptides targeting the FGF14:Nav1.6 PPI interface could be of pre-clinical value. In this work, we pharmacologically evaluated peptides derived from FGF14 that correspond to residues that are at FGF14’s PPI interface with the CTD of Nav1.6. These peptides, Pro-Leu-Glu-Val (PLEV) and Glu-Tyr-Tyr-Val (EYYV), which correspond to residues of the β12 sheet and β8-β9 loop of FGF14, respectively, were shown to inhibit FGF14:Nav1.6 complex assembly. In functional studies using whole-cell patch-clamp electrophysiology, PLEV and EYYV were shown to confer differential modulation of Nav1.6-mediated currents through mechanisms dependent upon the presence of FGF14. Crucially, these FGF14-dependent effects of PLEV and EYYV on Nav1.6-mediated currents were further shown to be dependent on the N-terminal domain of FGF14. Overall, these data suggest that the PLEV and EYYV peptides represent scaffolds to interrogate the Nav1.6 channel macromolecular complex in an effort to develop targeted pharmacological modulators.

## Introduction

Voltage-gated Na^+^ (Nav) channels are responsible for the initiation and propagation of action potentials in excitable cells (Catterall, 2012). This functionality is largely conferred via the pore-forming α subunit of Nav channels, of which nine different isoforms (Nav1.1-Nav1.9) have been described. In addition to molecular differences among these nine isoforms of the Nav channel α subunit, they also diverge with respect to their tissue distribution. Specifically, Nav1.1-Nav1.3 and Nav1.6 are expressed in the central nervous system (CNS); Nav1.4 is expressed in skeletal muscle; Nav1.5 is expressed in cardiac muscle; and Nav1.7-Nav1.9 are expressed in the peripheral nervous system (PNS) (Goldin et al., 2000; Yu and Catterall, 2003; Catterall et al., 2005; Chahine et al., 2008; Savio-Galimberti et al., 2012; Dib-Hajj et al., 2015). Given this ubiquitous expression throughout the body, it is unsurprising that mutations to specific Nav channel isoforms give rise to an array of disease-states including autism spectrum disorder (Sanders et al., 2012; Tavassoli et al., 2014), ataxia (Savio-Galimberti et al., 2012), Dravet syndrome, cognitive impairment, epilepsy (Claes et al., 2001; Mantegazza et al., 2005, 2010; Catterall et al., 2010; Volkers et al., 2011; Schaefer et al., 2013; Oyrer et al., 2018), Brugada syndrome (Probst et al., 2009), pain-related syndromes (Woods et al., 2015; Wright et al., 2016), primary erythromelalgia (Tang et al., 2015), paroxysmal extreme pain disorder (Dib-Hajj et al., 2009; Lampert et al., 2010); and cardiac arrhythmias (Wang et al., 1995; Musa et al., 2015).

Given their essential role in regulating physiology throughout the body, Nav channels have historically been a traditional target for drug development. Unfortunately, current therapeutics targeting Nav channels bind to structural motifs of the α subunit that display high amino acid sequence homology among the nine Nav channels isoforms, which results in these therapeutics lacking isoform selectivity and giving rise to deleterious off-target side effects due to modulation of off-target Nav channel isoforms (Catterall and Swanson, 2015). To address this challenge, the identification of novel Nav channel drug-binding sites is a necessary pre-requisite to identify therapeutics with improved selectivity (Dvorak et al., 2021).

Among structural components of the Nav channel that could be pharmacologically targeted to achieve improved selectivity, C-terminal domains (CTD) of Nav channels stand out as promising surfaces to target, as they display amino acid sequence divergence among isoforms that enables structurally and functionally specific protein:protein interactions (PPI) with auxiliary proteins (Lou et al., 2005; Laezza et al., 2007, 2009; Tseng et al., 2007; Wang et al., 2011; Pitt and Lee, 2016; Effraim et al., 2019; Lu et al., 2020). In the central nervous system (CNS), one salient example of such a PPI occurs between the CTD of Nav1.6 and its auxiliary protein fibroblast growth factor 14 (FGF14) (Liu et al., 2003; Lou et al., 2005; Goetz et al., 2009; Laezza et al., 2009; Ali et al., 2016, 2018; Hsu et al., 2016; Singh et al., 2020; Wadsworth et al., 2020). Specifically, this PPI regulates the transient and resurgent Na+ currents of neurons through a mechanism thought to depend upon the N-terminus of FGF14 (Yan et al., 2014; White et al., 2019), as well as the action potential (AP) firing of neurons in clinically relevant brain regions, including the nucleus accumbens (NAc) (Ali et al., 2018) and hippocampus (Hsu et al., 2016). Translationally, perturbation of this PPI is increasingly being associated with a myriad of neurologic and neuropsychiatric disorders (Di Re et al., 2017; Paucar et al., 2020), highlighting its potential clinical relevance as a pharmacological target.

To guide drug discovery efforts targeting the PPI interface between FGF14 and the CTD of Nav1.6, we previously developed and interrogated a homology model of the PPI interface to identify putative clusters of amino acids central to assembly of the complex (Ali et al., 2014, 2016). These investigations identified the Phe-Leu-Pro-Lys (FLPK) and Pro-Leu-Glu-Val (PLEV) motifs on the β12 sheet of FGF14, and the Glu-Tyr-Tyr-Val (EYYV) motif on the β8-β9 loop of FGF14, as being putatively essential for FGF14:Nav1.6 complex assembly (Ali et al., 2014, 2016). To investigate if short peptides derived from these clusters of amino acids could exert functionally relevant modulation of the Nav1.6 channel macromolecular complex, we previously reported our pharmacological evaluation of the FLPK tetrapeptide (Singh et al., 2020). In that work, we showed that FLPK inhibited FGF14:Nav1.6 complex assembly, reversed FGF14-mediated regulatory effects on Nav1.6 channel activity and affected neuronal excitability of MSNs of the NAc (Singh et al., 2020). Based upon this premise, we sought in the current work to investigate the modulatory effects of PLEV and EYYV on the Nav1.6 channel macromolecular complex. By employing the split-luciferase complementation assay (LCA) and whole-cell patch clamp electrophysiology, we show that these two short peptides derived from FGF14 confer inhibitory effects on FGF14:Nav1.6 complex assembly. Correspondingly, both peptides confer functionally relevant modulation of Nav1.6 channel activity in a manner dependent on the N-terminal domain of FGF14. Overall, this study demonstrates that short peptides derived from “hot spot” (London et al., 2010, 2013) of PPI interfaces could serve as innovative probes to guide drug discovery efforts.

## Material and Methods

### Materials

D-luciferin (Gold Biotechnology, St. Louis, MO) was prepared as a 30 mg/ml stock solution in phosphate-buffered saline (PBS), and stored at −20 C. PLEV and EYYV peptides were synthesized with 98% purity from Zhejiang Ontores Biotechnologies Co. (Yuhang District, Hangzhou, Zhejiang, China). Peptides were reconstituted in 100% dimethyl sulfoxide (DMSO) as 50 mM stock solutions and stored −20°C.

### Plasmid Constructs

Plasmid constructs used in this study were derived from the following clones: human FGF14-1b isoform (accession number: NM_175929.2); human Nav1.6 (accession number: NM_014191.3). The CLuc-FGF14, CD4-Nav1.6-NLuc constructs and the pcDNA3.1 vector (Invitrogen, Carlsbad, CA) were engineered and characterized as previously described (Goetz et al., 2009; Shavkunov et al., 2012; Shavkunov et al., 2013; Shavkunov et al., 2015; Ali et al., 2016, 2018; Wadsworth et al., 2019, 2020; Singh et al., 2020). The plasmid pGL3 expressing full-length Firefly (*Photinus pyralis*) luciferase was a gift from P. Sarkar (Department of Neurology, UTMB). To perform electrophysiological studies, FGF14-GFP and FGF14-ΔNT-GFP (64–252 amino acid residues) were sub-cloned into the GFP plasmid pQBI-fC2 (Quantum Biotechnology Inc., Montreal, Canada) as previously described (Singh et al., 2020).

### Homology Model-Based Docking of PLEV and EYYV to FGF14

The homology model-based docking was run with Schrödinger Small-Molecule Drug Discovery Suite using the FGF14 chain of a previously described FGF14:Nav1.6 homology model (Ali et al., 2016; Singh et al., 2020). The structure for protein was prepared by using Protein Prepared Wizard and peptide fragments (PLEV or EYYV containing N-terminal acetylation and C-terminal amidation) were prepared with LigPrep and further initial lowest energy conformation was obtained. The grid box coordinates, grid generation, docking employment, docking poses were analyzed as previously described (Singh et al., 2020).

### Cell Culture

HEK293 cells were cultured in a 1:1 mixture of Dulbecco’s Modified Eagle Medium (DMEM) with 1 g/L glucose and F-12 (Invitrogen, Carlsbad, CA, United States) that was additionally supplemented with 10% fetal bovine serum, 100 units/ml of penicillin, and 100 µg/ml streptomycin (Invitrogen). HEK293 cells stably expressing CLuc-FGF14 and CD4-Nav1.6-NLuc constructs were maintained similarly except for the addition of 500 μg/ml G418 and 100 ug/ml puromycin (Invitrogen) to maintain stable expression. This cell line was developed and characterized in previous studies and is hereafter coded as “Clone V” cells (Wadsworth et al., 2019). Cells were grown at 37°C. The HEK293 cells stably expressing human Na_v_1.6 channels have previously been described (Ali et al., 2016, 2018; Singh et al., 2020; Wadsworth et al., 2020). For transient transfections, the Lipofectamine 2000 protocol was followed (Invitrogen, Waltham, MA, United States), and the amount of cDNA used was 1 µg for each. For whole-cell, patch-clamp recordings HEK293-Nav1.6 cells were washed and replated at very low density prior to incubating the cells with peptides for recordings (Ali et al., 2016, 2018; Wadsworth et al., 2019, 2020; Singh et al., 2020).

### In Cell Split Luciferase Assay

HEK293 cells stable expressing CLuc-FGF14 and CD4-Nav1.6-NLuc (Clone-V) were grown for 24–48 h. Clone-V cells were detached using TrypLE (Gibco, Waltman, MA, United States), triturated in medium, and seeded in white, clear-bottom CELLSTAR μClear® 96-well tissue culture plates (Greiner Bio-One) at ∼0.8 × 105 cells per well in 200 μl of medium. The cells were treated for 12 h in a growth medium supplemented with 100 μl of serum-free, phenol red–free DMEM/F12 medium (Invitrogen) containing PLEV or EYYV (1–250 μM). The final concentration of DMSO was maintained at 0.5% for all wells. Following 12 h incubation at 37°C, the luminescence reaction was initiated by injection of 100 μl substrate solution containing 1.5 mg/ml of D-luciferin dissolved in PBS (final concentration = 0.75 mg/ml) by the Synergy™ H4 Multi-Mode Microplate Reader (BioTek). LCA readings were performed at 2 min intervals for 20–30 min, integration time 0.5 s, while cells were maintained at 37°C throughout the measurements. Detailed LCA method can be found in previous studies (Shavkunov et al., 2012; Ali et al., 2014, 2016, 2018; Hsu et al., 2015; Wadsworth et al., 2019, 2020; Singh et al., 2020).

### Electrophysiology in Heterologous Cells

Whole-cell voltage-clamp recordings in heterologous cell systems were performed as previously described (Ali et al., 2016, 2018; Singh et al., 2020; Wadsworth et al., 2020). The HEK293 cells cultured as described above were dissociated using TrypLE and re-plated at very low density onto glass coverslips. HEK293 cells were then allowed at least 3–4 h for attachment before coverslips were transferred to the recording chamber. The recording chamber was filled with a freshly prepared extracellular recording solution comprised of: 140 mM NaCl; 3 mM KCl; 1 mM MgCl_2_; 1 mM CaCl_2_; 10 mM HEPES; and 10 mM glucose (pH = 7.3; all salts purchased from Sigma-Aldrich, St. Louis, MO, United States). For control recordings, DMSO was added to the extracellular solution to obtain a final concentration of 0.1%. For the peptide conditions a PLEV and EYYV peptides were added to the extracellular solution to obtain their final concentrations. Cells were pre-incubated for at least 30 min in either DMSO or peptides containing extracellular solutions prior to the recordings. The pipettes were filled with an intracellular solution: 130 mM CH_3_O_3_SCs; 1 mM EGTA; 10 mM NaCl; and 10 mM HEPES (pH = 7.3; all salts purchased from Sigma-Aldrich). The glass pipettes (Harvard Apparatus, Holliston, MA, United States) with a resistance of 3–5 MΩ were fabricated using a PC-100 vertical Micropipette Puller (Narishige International Inc., East Meadow, NY, United States). Recordings were obtained using an Axopatch 700B or 200B amplifier (Molecular Devices, Sunnyvale, CA, United States). Membrane capacitance and series resistance were estimated using the dial settings on the amplifier, and capacitive transients and series resistances were compensated by 70–80%. Data acquisition and filtering occurred at 20 and 5 kHz, respectively, before digitization and storage. Clampex 9.2 software (Molecular Devices) was used to set experimental parameters, and electrophysiological equipment interfaced to this software using a Digidata 1,200 analog-digital interface (Molecular Devices). Analysis of electrophysiological data was performed using Clampfit 9 software (Molecular Devices) and GraphPad Prism 7 software (La Jolla, CA, United States). After GΩ seal formation and entry into the whole-cell configuration, the voltage-clamp protocols were employed such as the current-voltage (IV) protocol which entailed voltage-steps from −100 mV to +60 mV from a holding potential of −70 mV. The voltage-dependence of steady-state inactivation was calculated using a paired-pulse protocol during which, from the holding potential, cells were stepped to varying test potentials between −20 mV and +20 mV prior to a test pulse to −20 mV.

Current densities were obtained by dividing Na^+^ current (*I*_Na_) amplitude by membrane capacitance. Current-voltage relationships were then assessed by plotting current density as a function of applied voltage. Tau (τ) of fast inactivation was calculated by fitting the decay phase of currents at the -10 mV voltage step with a one-term exponential function. To assess voltage-dependence of activation, conductance (G_Na_) was first calculated using the following equation:

$$G_{Na}=\;\frac{I_{Na}}{\left(V_{m}-E_{rev}\right)}$$

where *I*_Na_ is the current amplitude at voltage V_m_, and E_rev_ is the Na^+^ reversal potential. Activation curves were then generated by plotting normalized G_Na_ as a function of the test potential. Data was then fitted with the Boltzmann equation to determine V_1/2_ of activation using the following equation:

$$\frac{G_{Na}}{G_{Na,\;max}}=\;1+\;e^{V_{a}-E_{m}/k}\;$$

where G_Na,max_ is the maximum conductance, V_a_ is the membrane potential of half-maximal activation, E_m_ is the membrane voltage, and k is the slope factor. For steady-state inactivation, normalized current amplitude (*I*_Na_/*I*_Na,max_) at the test potential was plotted as a function of pre-pulse potential (V_m_) and fitted using the Boltzmann equation:

$$\frac{I_{Na}}{I_{Na,max}}=\;\frac{1}{1+\;e^{V_{h}-E_{m}/k}}$$

where V_h_ is the potential of half-maximal inactivation, E_m_ is the membrane voltage, and k is the slope factor.

To study long-term inactivation (LTI), a four-sweep protocol consisting of four 20 ms duration, 0 mV depolarizing pulses separated by 40 ms duration −90 mV interpulse recovery phases from a -90 mV holding potential was employed (Dover et al., 2010; Barbosa et al., 2017; Liu et al., 2019; Tapia et al., 2020).

Cumulative inactivation was examined by applying a 2 ms test pulse to −10 mV 20 times at frequency10 Hz from a holding potential of −80 mV. Current responses were normalized to the first recorded pulse and the currents at the 20^th^ pulses were compared (Laezza et al., 2009; Effraim et al., 2019).

### Data Analysis

Results are expressed as mean ± standard error of the mean (SEM). Except where otherwise noted, statistical significance was determined using a Student’s *t*-test or one way ANOVA Tukey’s multiple comparisons test, comparing cells treated with vehicle (DMSO) or PLEV and EYYV, with *p* < 0.05 being considered statistically significant. The analysis was performed by using GraphPad Prism^R^ (La Jolla, CA) software.

## Results

### Homology Model-Based Docking of PLEV and EYYV to the Protein-Protein Interaction Interface Between FGF14 and the CTD of Nav1.6

**FIGURE 1:**
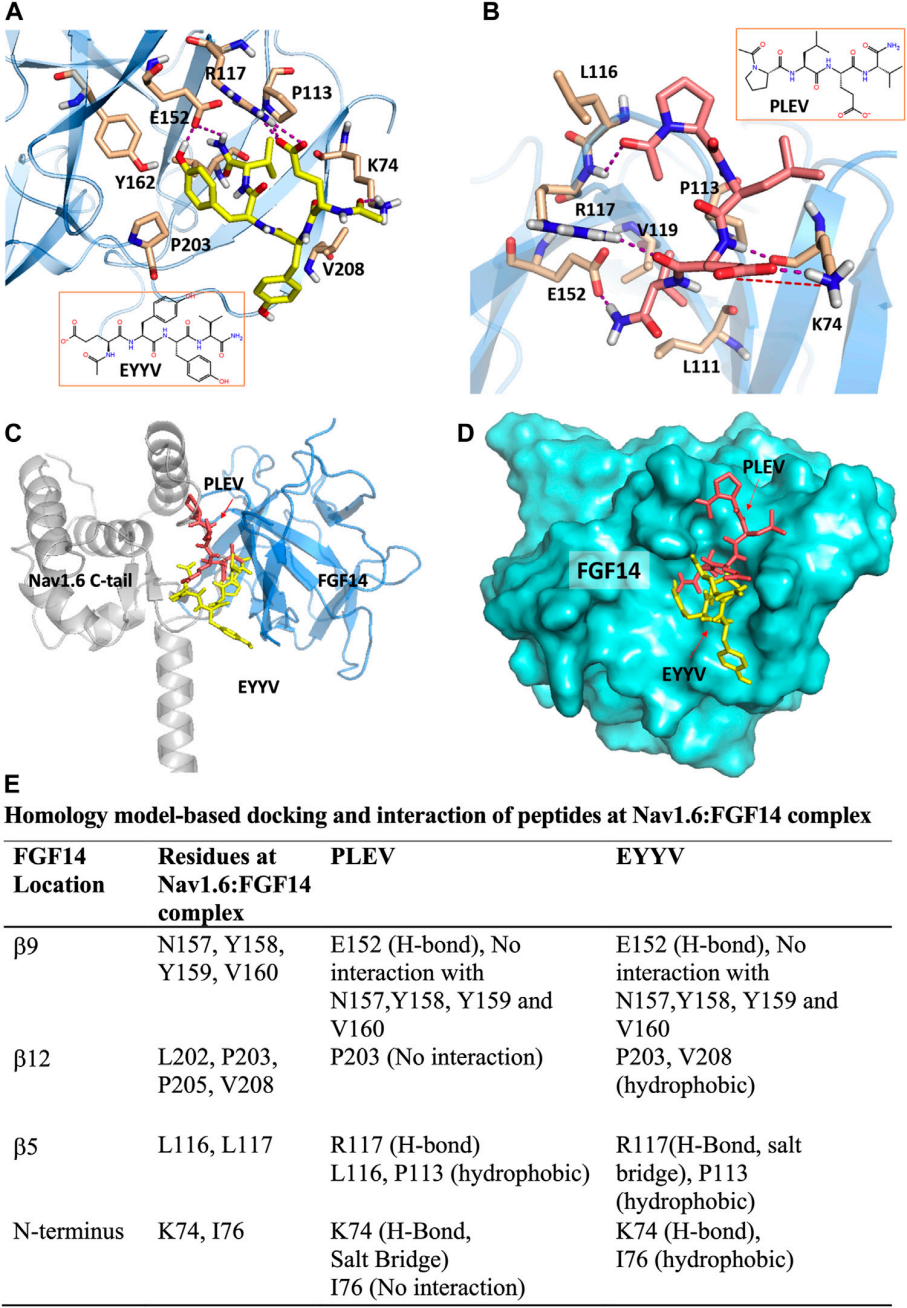
PLEV and EYYV docking to the FGF14:Nav1.6 complex (modified Singh et al., 2020. Physiol Rep. PMID: 32671946). **(A,B)** Representation of PLEV (pink-orange) and EYYV (yellow) peptide fragments docked into the FGF14 chain of the FGF14:Nav1.6 C-terminal tail homology model. FGF14 has depicted as sky blue ribbons. Key interaction residues are highlighted as stick representations. H-bonds are shown as purple dotted lines, salt bridges are shown as red dotted lines. Residues shown in the map are within 4 Å cut-off. **(C)** Overlay of PLEV (pink-orange) and EYYV (yellow) docked poses and FGF14. Nav1.6 C-terminal tail complex homology model. The FGF14 chain is depicted as sky blue ribbons and the Nav1.6 C-terminal tail is highlighted as gray ribbons. The overlay analysis demonstrated the peptides bound with FGF14 to the different locations at the FGF14:Nav1.6 C-tail protein-protein interaction (PPI) interface. **(D)** Surface representation of PLEV (pink-orange) and EYYV (yellow) peptide fragments docked pose overlay with FGF14 (cyan). **(E)** table representing the Key interacting residues of FGF14:Nav1.6 C-terminal tail PPI with PLEV and EYYV.

The peptides used in this study were developed based upon segments of FGF14 that are known to be at the interface of the FGF14:Nav1.6 macromolecular complex. PLEV corresponds to an amino acid sequence on the β12 sheet of FGF14, and EYYV corresponds to a sequence on the *β*8-*β*9 loop of FGF14 (Ali et al., 2014). These peptides were docked to a homology model of the PPI interface between FGF14 and the CTD of Nav1.6. (Ali et al., 2016, 2018; Dvorak et al., 2020). In our previous study (Singh et al., 2020) we predicted that PLEV and EYYV were interacting toward the periphery of FGF14 at the β5 strand and N-terminus. We further showed the interactions of PLEV and EYYV with key residues at the FGF14:Nav1.6 PPI interface (modified Figure 1, Singh et al., 2020). The molecular docking of PLEV and EYYV revealed predicted interactions with residues at the FGF14:Nav1.6 PPI interface suggestive of the ability to disrupt FGF14:Nav1.6 complex assembly. For example, PLEV and EYYV displayed conserved interactions with R117 on the *β*5 strand of FGF14, a residue that is a crucial constituent of the core domain of FGF14 and is essential for enabling FGF14-mediated regulatory effects on transient *I*_Na_ and resurgent *I*_Na_ (Yan et al., 2014). Additionally, PLEV and EYYV displayed conserved interactions with K74 of the N-terminus of FGF14, which could be predictive of these peptides displaying functional activity due to the central role of the N-terminus of FGF14 in conferring modulatory effects on Nav channel inactivation and resurgent *I*_Na_ (Laezza et al., 2007; Yan et al., 2014; White et al., 2019). Despite these conserved interactions, PLEV and EYYV also displayed divergent interactions with residues at the FGF14:Nav1.6 PPI interface, such as with P203, which could be suggestive of differential effects on Nav1.6 channel activity. Based upon these predicted interactions with residues at the FGF14:Nav1.6 PPI interface *in silico*, both PLEV and EYYV were further investigated in biological systems to interrogate potential modulatory effects on the Nav1.6 channel macromolecular complex.

### In-Cell Testing of PLEV and EYYV Using the Split Luciferase Complementation Assay

**FIGURE 2:**
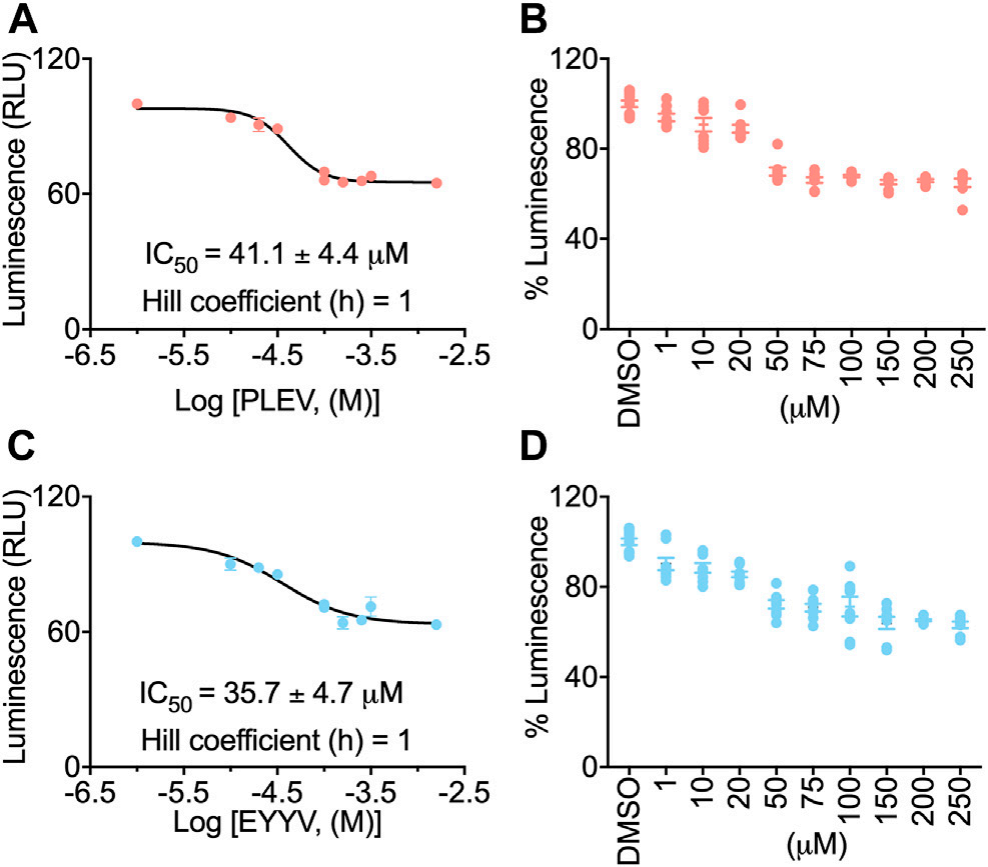
In-cell evaluation of dose-dependent effects of the PLEV and EYYV tetrapeptides on FGF14:Nav1.6 complex assembly using the LCA. **(A)** Percent luminescence plotted as a function of log concentration of PLEV to characterize the dose-dependent effects of the tetrapeptide on FGF14:Nav1.6 complex assembly. **(B)** Bar graph showing dose-response as depicted in panel A. **(C)** Percent luminescence plotted as a function of log concentration of EYYV to characterize the dose-dependent effects of the tetrapeptide on FGF14:Nav1.6 complex assembly. **(D)** Bar graph showing dose-response as depicted in panel B. PLEV or EYYV concentrations were used (1, 10, 25, 50, 75, 100, 150, and 250 μM; *n* = 8 per concentration) for dose response. Data were normalized to per plate wells treated with 0.5% DMSO, and non-linear regression curve was performed using GraphPad Prism to calculate IC Data represented as mean ± SEM.

To evaluate the hypothesis that PLEV and/or EYYV could modulate FGF14:Nav1.6 complex assembly, an in-cell assay was used with a HEK293 cell line (hereafter coded as “Clone V” cells) stably expressing both CLuc-FGF14 and CD4-Nav1.6-NLuc (Shavkunov et al., 2012; Shavkunov et al., 2013; Shavkunov et al., 2015; Ali et al., 2016, 2018; Wadsworth et al., 2019, 2020; Dvorak et al., 2020, 2021; Singh et al., 2020; Wang et al., 2020). In this assay, upon binding of FGF14 to the Nav1.6 C-terminal tail, there is reconstitution of the NLuc and CLuc halves of the luciferase enzyme, which produces luminescence in the presence of substrate D-luciferin (Figures 2A,D). Employing this assay in the current study, Clone V cells were seeded into 96 well plates and then treated with various concentrations (1–250 µM) of PLEV, EYYV, or 0.5% DMSO. After incubation, luciferin was dispensed, and the luminescence observed in each well was recorded. The max luminescence observed in each well was then normalized to the average max luminescence observed in per plate control wells. These investigations revealed that PLEV and EYYV displayed dose-dependent inhibitory effects on FGF14:Nav1.6 complex assembly. Specifically, sigmoidal fitting of the dose response curve for PLEV and EYYV revealed IC_50_ values of 41.1 ± 4.4 µM and 35.7 ± 4.7 µM, respectively. (Figures 2B,C,E,F). Having demonstrated their inhibitory effects on FGF14:Nav1.6 complex assembly, the functional effects of these peptides on Nav1.6 channel activity were subsequently assessed using whole-cell patch-clamp electrophysiology in heterologous cells.

### PLEV and EYYV Differentially Modulate Peak Transient INa in the Presence of FGF14

**FIGURE 3:**
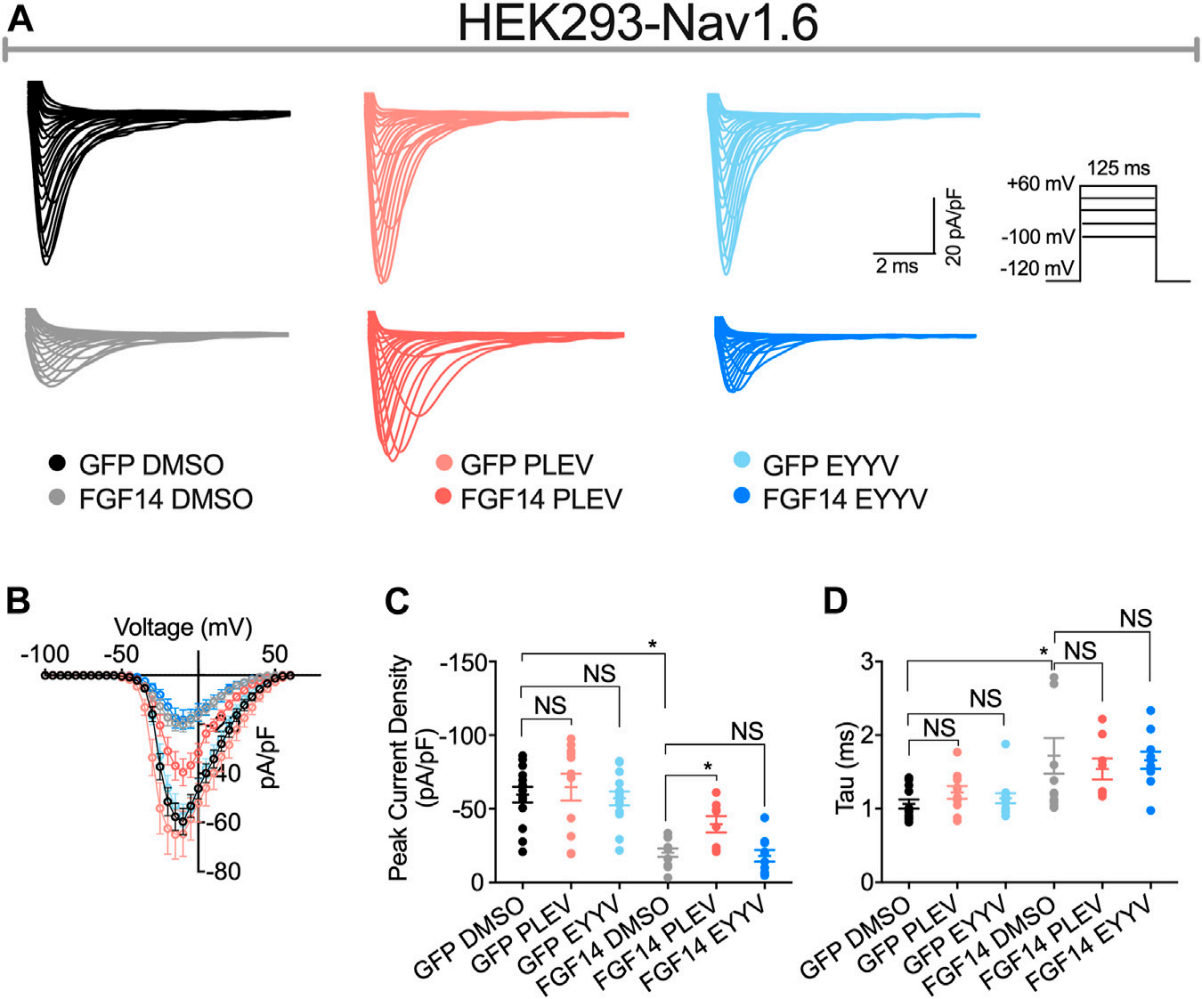
PLEV and EYYV differentially modulate peak transient INa density in the presence of FGF14. **(A)** Representative traces of transient Na+ currents elicited by HEK293-Nav1.6 cells co-expressing either GFP of FGF14-GFP that had been treated with 0.1% DMSO, 50 µM PLEV, or 50 µM EYYV in response to voltage-steps from −100 mV to +60 mV from a holding potential of – 70 mV (inset). **(B)** Current-voltage relationships for experimental groups described in (A). (C,D) Comparison of peak transient INa density **(C)** and tau (τ) of fast inactivation **(D)** for the indicated experimental groups. Data are mean ± SEM. Significance was assessed using a one-way ANOVA Tukey’s multiple comparisons test. NS = non-significant; *, *p* at least < 0.05 (detailed *p* values and summary of results are reported in Table 1).

**FIGURE 4:**
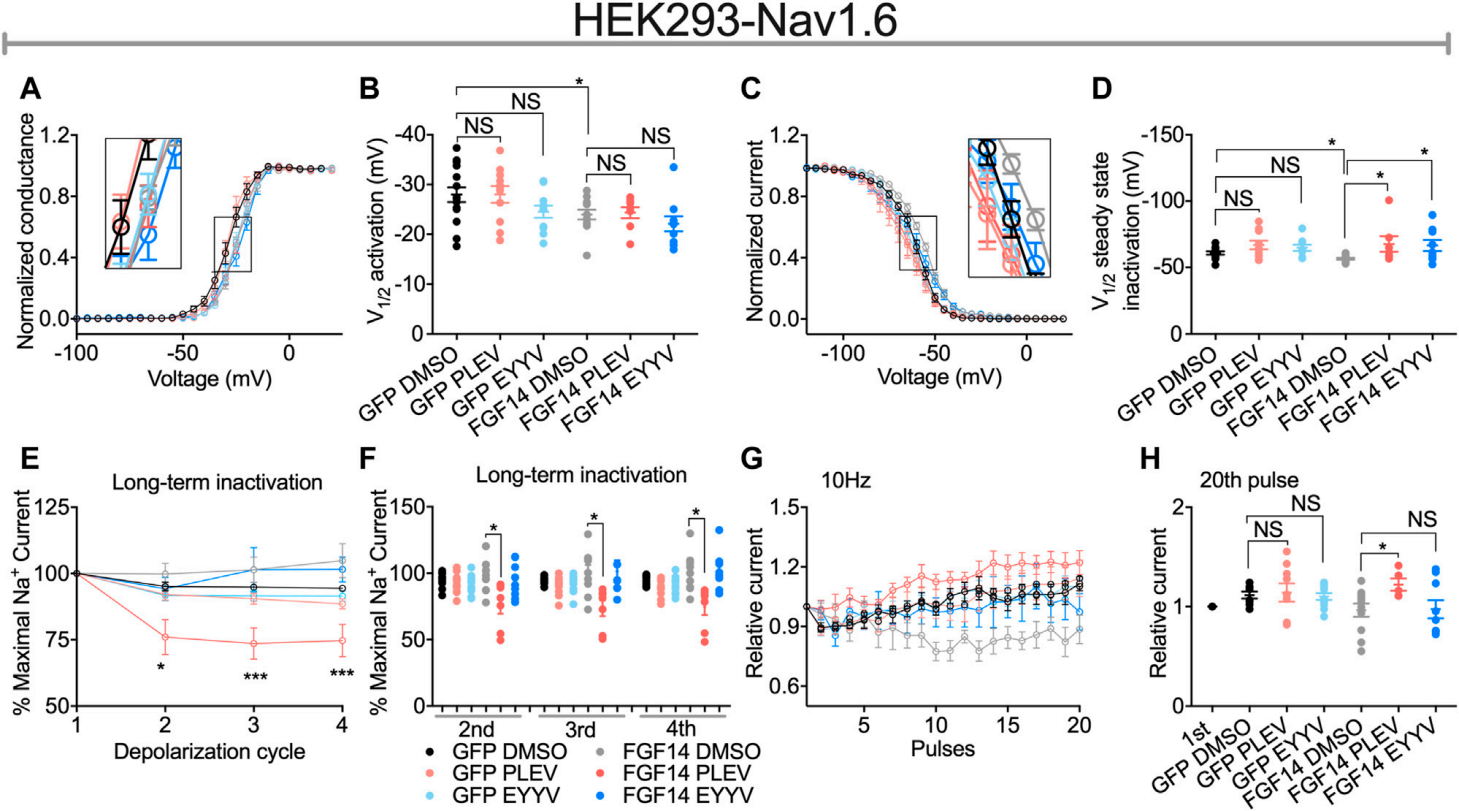
Functional regulation of Nav1.6-mediated currents by PLEV and EYYV. **(A)** Conductance-voltage relationships for Nav1.6 cells co-expressing GFP or FGF14-GFP treated with 0.1% DMSO, 50 µM PLEV and 50 µM EYYV. **(B)** Comparison of V1/2 of activation between the indicated experimental groups. **(C)** Normalized current as a function of membrane potential to characterize the effects of 0.1% DMSO, 50 µM PLEV, and 50 µM EYYV on steady-state inactivation. **(D)** Comparison of V1/2 of steady-state inactivation between the indicated experimental groups. **(E)** Ratio maximal INa plotted as a function of depolarization cycle to characterize the effects of 0.1% DMSO, 50 µM PLEV, or 50 µM EYYV on entry of Nav1.6 channels into long-term inactivation (LTI) in the presence and absence of FGF14. Cells were subjected to four 0 mV 16 ms depolarizations separated by −90 mV 40 ms recovery intervals. **(F)** Bar graph represented 2nd, 3rd, and 4th pulse of LTI. (G,H) Characterization of cumulative inactivation of Nav1.6 channels in the presence or absence of FGF14 exposed to 0.1% DMSO, 50 µM PLEV, or 50 µM EYYV. It was examined by applying a 2 ms test pulse to −10 mV, 20 times at frequency10 Hz from a holding potential of −80 mV. Data are mean ± SEM. Significance was assessed using a one-way ANOVA Tukey’s multiple comparisons test. NS = non-significant; *, *p* value at least < 0.05 (detailed *p* values and summary of results are reported in Tables 1, 3).

Having determined the in-cell potency with which PLEV and EYYV inhibit FGF14:Nav1.6 complex assembly, we next used electrophysiology to evaluate whether PLEV or EYYV displayed modulatory effects on Nav1.6-mediated currents. These recordings were performed in HEK293 cells stably expressing Nav1.6 and transiently transfected with GFP (Nav1.6-GFP) or FGF14-GFP (Nav1.6-FGF14-GFP). The cells were incubated for at least 30 min with 0.1% DMSO or either one of the two tetrapeptides at 50 µM final concentration in a static bath before recording. After incubation, the modulatory effects of PLEV and EYYV on Nav1.6-mediated currents were assessed using whole-cell patch-clamp recordings (Figures 3, 4 and Table 1). In agreement with previous studies (Ali et al., 2016, 2018; Singh et al., 2020; Wadsworth et al., 2020), expression of FGF14-GFP suppressed the Nav1.6-mediated peak transient Na^+^ current (*I*_Na_) density (−20.58 ± 3.03 pA/pF, *n* = 12 vs −59.63 ± 5.33 pA/pF, *n* = 15; *p* < 0.001; one-way ANOVA Tukey’s multiple comparison test; Figures 3A,B and Table 1). In the presence of PLEV, the FGF14-mediated suppression of Nav1.6 current was partially reversed compared to control (FGF14-GFP + PLEV: −39.51 ± 5.54 pA/pF, *n* = 8; FGF14-GFP + DMSO: −20.58 ± 3.03 pA/pF, *n* = 12; *p* = 0.0428; one-way ANOVA Tukey’s multiple comparison test). Conversely, EYYV failed to modulate the FGF14-mediated regulatory effects on Nav1.6 peak transient *I*_Na_ density as compared to control (FGF14-GFP + EYYV: −18.1 ± 3.92 pA/pF, *n* = 10; FGF14-GFP + DMSO: −20.58 ± 3.03 pA/pF, *n* = 12; *p* = 0.9890; Figures 3A,B and Table 1). However, both PLEV and EYYV had no effect when tested in the channel alone experimental group (HEK-Nav1.6-GFP), as the Nav1.6-mediated transient peak *I*_Na_ density was not significantly changed (PLEV: −64.66 ± 9.1 pA/pF, *n* = 11; EYYV: −57.09 ± 4.66, *n* = 14) compared to DMSO (−59.63 ± 5.33 pA/pF, *n* = 15; Figures 3A,B and Table 1). Consistent with previous studies (Ali et al., 2016, 2018), co-expression of FGF14 with Nav1.6 increased the decay time constant (τ) of fast inactivation (1.72 ± 0.24 ms, *n* = 14 versus 1.06 ± 0.06 ms, *n* = 12 for FGF14-GFP + DMSO versus GFP + DMSO, respectively; *p* < 0.05). Despite this FGF14-mediated regulatory effect on the entry of Nav1.6 channels into fast inactivation, neither PLEV nor EYYV displayed effects on τ in the presence or absence of FGF14 (Figure 3D; Table 1).

**TABLE 1:** Nav1.6 currents in the presence of FGF14 and tetrapeptides PLEV, EYYV.

Condition	Peak density	Activation	K_act_	Steady-state inactivation	K_inact_	Tau (τ)
	pA/pF	mV	mV	mV	mV	Ms
GFP DMSO	−59.63 ± 5.33 (14)	−27.98 ± 1.48 (15)	5.23 ± 0.55 (12)	−60.93 ± 1.3 (13)	5.88 ± 0.59 (13)	1.06 ± 0.06 (12)
GFP PLEV	−64.66 ± 9.1 (11)	−28.01 ± 1.67 (11)	3.97 ± 0.57 (11)	−66.9 ± 3.25 (10)	5.90 ± 0.87 (10)	1.48 ± 0.25 (11)
GFP EYYV	−57.09 ± 4.66 (14)	−24.56 ± 1.20 (11)	4.37 ± 0.40 (11)	−64.93 ± 2.31 (9)	6.57 ± 0.37 (9)	1.14 ± 0.06 (13)
FGF14-GFP DMSO	−20.58 ± 3.03 (11)^a^	−24.21 ± 1.02 (12)^b^	6.66 ± 0.84 (12)	−56.67 ± 0.69 (14)^c^	6.52 ± 0.74 (14)	1.72 ± 0.24 (14)^d^
FGF14-GFP PLEV	−39.51 ± 5.54 (8)^e^	−24.35 ± 1.10 (8)	4.55 ± 0.39 (8)	−67.66 ± 5.85 (7)^f^	7.89 ± 0.90 (7)	1.54 ± 0.14 (8)
FGF14-GFP EYYV	−18.1 ± 3.92 (10)	−22.11 ± 1.49 (10)	5.97 ± 0.69 (10)	−66.59 ± 4.16 (9)^g^	6.91 ± 0.65 (9)	1.66 ± 0.12 (10)

Data are mean ± SEM. ns, non-significant.

a *p* < 0.0001, one-way ANOVA post hoc Tukey’s multiple comparisons test compared to GFP DMSO.

b *p* = 0.0428, Student’s *t-*test compared to GFP DMSO.

c *p* = 0.007, Student’s *t-*test compared to GFP DMSO.

d *p* = 0.0337, one-way ANOVA post hoc Tukey’s multiple comparisons test compared to GFP DMSO.

e *p* = 0.0258, one-way ANOVA post hoc Tukey’s multiple comparisons test compared to GFP DMSO.

f *p* = 0.0497, one-way ANOVA post hoc Tukey’s multiple comparisons test compared to FGF14-GFP DMSO.

g *p* = 0.0084, Student’s *t-*test compared to FGF14-GFP DMSO.

### PLEV and EYYV Display Convergent Modulatory Effects on Steady-State Inactivation, But Divergent Modulatory Effects on Long-Term and Cumulative Inactivation

Consistent with previous investigations (Ali et al., 2016, 2018; Singh et al., 2020), co-expression of FGF14 with Nav1.6 resulted in depolarizing shifts in the voltage-dependence of Nav1.6 channel activation (−24.21 ± 1.02 mV, *n* = 12 versus 27.98 ± 1.48 mV, *n* = 14 for FGF14-GFP + DMSO and GFP + DMSO, respectively; *p* < 0.05; Figures 4A,B and Table 1) and Nav1.6 channel steady-state inactivation (−56.67 ± 0.69 mV, *n* = 14 versus −60.93 ± 1.3 mV, *n* = 13 for FGF14-GFP + DMSO and GFP + DMSO, respectively; *p* < 0.05; Figures 4C,D and Table 1). Whereas neither peptide displayed modulatory effects on the voltage-dependence of activation (Figures 4A,B and Table 1), both peptides reversed the FGF14-mediated depolarizing shift in Nav1.6 channel steady-state inactivation (Figures 4C,D). Similar to the results shown in Figure 3, both peptides displayed no effects in the absence of FGF14 on either Nav1.6 channel activation or steady-state inactivation.

In contrast to their conserved effects on Nav1.6 channel steady-state inactivation, PLEV and EYYV displayed divergent effects on long-term inactivation (LTI) and cumulative inactivation induced by applying a 2 ms test pulse to −10 mV 20 times at frequency of 10 Hz from a holding potential of −80 mV. Similar to results shown in Figure 3 and Figures 4A–D, both PLEV and EYYV displayed no effect on LTI or cumulative inactivation in the absence of FGF14 (Figures 4E–H and Table 1). Additionally, EYYV displayed no effects on LTI or cumulative inactivation in the presence of FGF14 (Figures 4E–H and Table 1). Notably, however, in the presence of FGF14, PLEV markedly altered the function of FGF14 as it pertains to its regulatory effects on LTI and cumulative inactivation. Specifically, treatment of HEK-Nav1.6 cells co-expressing FGF14 with PLEV resulted in an increased fraction of channels entering into LTI compared to HEK-Nav1.6 cells co-expressing FGF14 treated with vehicle (Figures 4E,F and Table 1). Paradoxically, treatment of HEK293-Nav1.6 cells co-expressing FGF14 with PLEV resulted in an increased number of available channels after repetitive stimulation, whereas treatment of HEK293-Nav1.6 cells co-expressing FGF14 with DMSO (control) resulted in roughly the same number of available channels before and after repetitive stimulation (Figures 4G,H and Table 1). Consistent with the results of the molecular modeling study shown in Figure 1, the results of these functional studies highlight conserved and divergent modulatory effects of PLEV and EYYV on the Nav1.6 channel macromolecular complex.

### Functional Effects of PLEV and EYYV on Nav1.6 Channel Activity Are Dependent Upon the Presence of the N-terminal Domain of FGF14

The role of the N-terminal domain of FGF14 in conferring its functional regulation of Nav channel activity is widely recognized (Lou et al., 2005; Laezza et al., 2007; Yan et al., 2014; White et al., 2019). According to current models of iFGF-mediated regulation of Nav channel activity, it is proposed that the core domain of FGF14 interacts with the CTD of Nav1.6, which resultantly orients the N-terminal domain of FGF14 such that it can occlude the internal mouth of the pore (Yan et al., 2014; White et al., 2019). Resultantly, both mutating residues of the core domain of FGF14 that enable its PPI with the CTD of Nav1.6 and truncation of the N-terminal domain of FGF14 have been shown to reverse FGF14-mediated regulation of Nav1.6 channel activity (Yan et al., 2014). Based upon this model of FGF14-mediated regulation of Nav1.6 channel activity, we next sought to investigate if PLEV and EYYV, which are derived from the core domain of FGF14, exert their modulatory effects on Nav1.6 channel activity through a mechanism dependent upon the N-terminal domain of FGF14. To do so, HEK293-Nav1.6 cells were transiently transfected with a cDNA construct corresponding to a FGF14 protein product with a truncated N-terminal domain (FGF14-ΔNT-GFP, 64–252 amino acid residues). Transiently transfected cells were then treated with 0.1% DMSO, 50 µM PLEV, or 50 µM EYYV, after which whole-cell patch-clamp electrophysiology was performed as described for Figure 3 and Figure 4.

**FIGURE 5:**
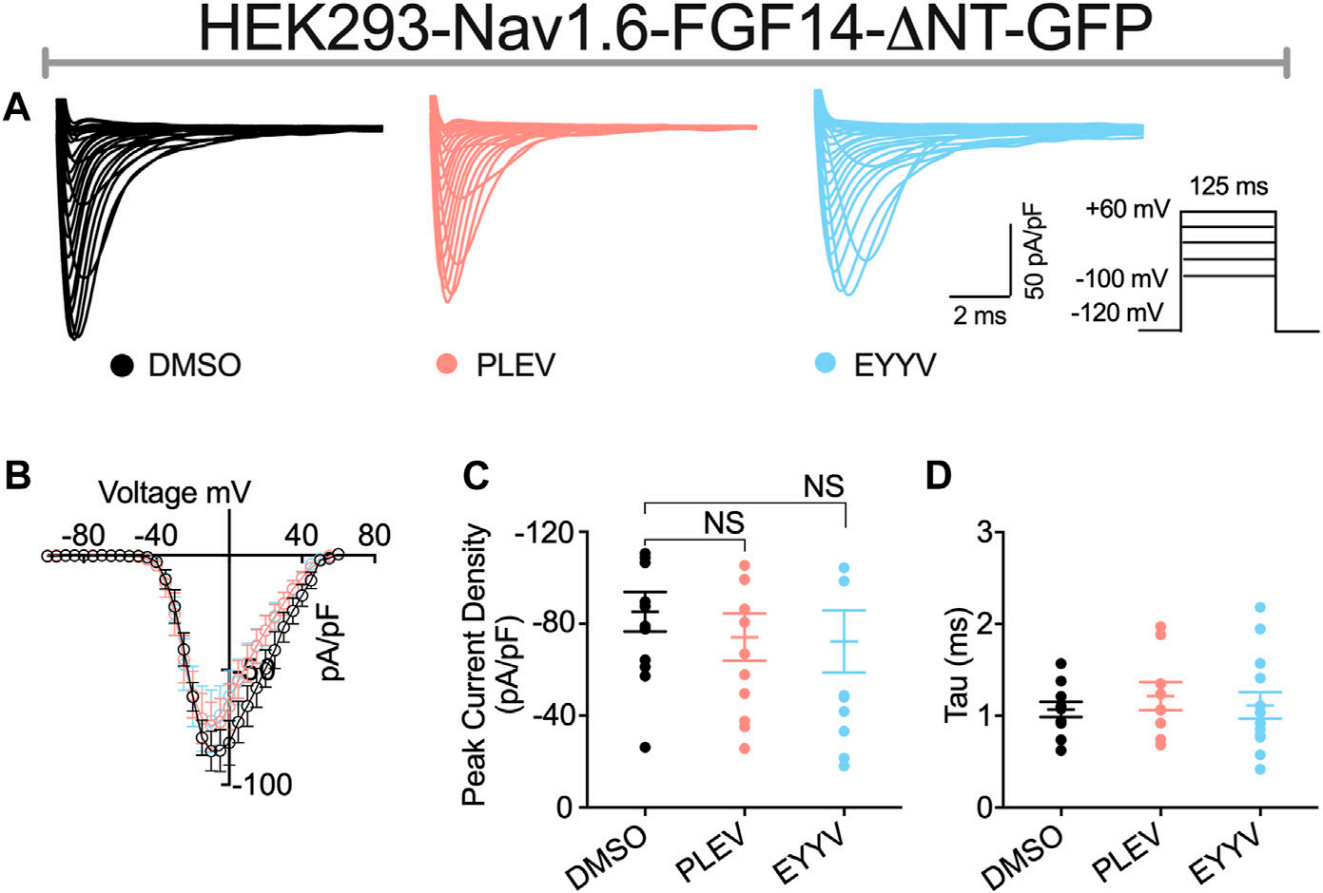
The N-terminal domain of FGF14 is required for functional activity of peptides on peak transient INa density. **(A)** Representative traces of transient Na+ currents elicited by HEK293-Nav1.6 cells co-expressing FGF14-ΔNT-GFP treated with 0.1% DMSO, 50 µM PLEV and 50 µM EYYV in response to voltage steps from −100 mV to +60 mV, holding potential of −70 mV (inset). **(B)** Current-voltage relationships for experimental groups described in **(A)**. **(C,D)** Comparison of peak transient INa density (C) and tau (τ) of fast inactivation (D) for the indicated experimental groups. Data are mean ± SEM. Significance was assessed using Student’s *t*-test comparing cells treated with 0.1% DMSO, 50 µM PLEV and 50 µM EYYV. NS = non-significant. Table summary of results is shown in Table 2.

In these studies, we observed the previously identified (Laezza et al., 2007; Singh et al., 2020) phenotype in which co-expression of FGF14-ΔNT with Nav1.6 resulted in a potentiation of Nav1.6 mediated peak transient *I*_Na_ density compared to co-expression of GFP (FGF14-ΔNT DMSO: −85.26 ± 8.59 pA/pF, *n* = 13 compared to GFP DMSO: −59.63 ± 5.33 pA/pF, *n* = 14; *p* = 0.0162, Student’s *t*-test; Figures 5A,B and Tables 1, 2). Unlike the effects of PLEV on peak transient *I*_Na_ density observed in the presence of FGF14 (see Figure 3), the peptide displayed no effects on this electrophysiological parameter when the N-terminal domain of FGF14 was truncated (Figures 5A–C). EYYV similarly displayed no effects on peak transient *I*_Na_ density in the presence of FGF14-ΔNT (Figures 5A–C). The lack of effects of both peptides on peak transient *I*_Na_ density in the presence of FGF14-ΔNT were accompanied by lack of effects on τ of fast inactivation (Figure 5D).

**TABLE 2:** Nav1.6 currents in the presence of FGF14-ΔNT and tetrapeptides PLEV, EYYV.

Condition	Peak density	Activation	K_act_	Steady-state Inactivation	K_inact_	Tau (τ)
	pA/pF	MV	mV	mV	mV	ms
FGF14-ΔNT-GFP DMSO	−85.26 ± 8.59 (13)	−23.13 ± 1.79 (13)	5.23 ± 0.55 (13)	−71.71 ± 3.54 (11)	5.88 ± 0.59 (11)	1.07 ± 0.08 (13)
FGF14-ΔNT-GFP PLEV	−74.19 ± 10.22 (12)	−25.65 ± 1.81 (11)	3.97 ± 0.57 (11)	−69.29 ± 3.58 (9)	5.90 ± 0.87 (9)	1.21 ± 0.15 (9)
FGF14-ΔNT-GFP EYYV	−72.25 ± 13.46 (11)	−22.96 ± 0.87 (11)	4.37 ± 0.40 (11)	−69.24 ± 2.22 (13)	6.57 ± 0.37 (13)	1.11 ± 0.14 (13)

Data are mean ± SEM; ns = nonsignificant.

**TABLE 3:** Nav1.6 channel LTI in the presence of FGF14 and tetrapeptides PLEV, EYYV.

Condition	LTI (% Maximal Na^+^ current)		
	2^nd^ Pulse	3^rd^ Pulse	4^th^ Pulse
GFP DMSO	95.12 ± 1.66 (11)	94.82 ± 0.83 (11)	94.41 ± 1.05 (11)
GFP PLEV	92.1 ± 2.22 (11)	90.48 ± 2.03 (11)	88.55 ± 1.87 (11)
GFP EYYV	91.69 ± 2.11 (11)	91.53 ± 2.01 (11)	91.48 ± 1.93 (11)
FGF14-GFP DMSO	99.77 ± 4.01 (11)	101.4 ± 4.74 (11)	104.9 ± 6.39 (11)
FGF14-GFP PLEV	76.06 ± 8.44 (7)^a^	73.56 ± 5.92 (7)^b^	74.69 ± 8.05 (7)^c^
FGF14-GFP EYYV	94.24 ± 4.33 (9)	101.4 ± 8.29 (9)	101.5 ± 6.16 (9)

Data are mean ± SEM. ns, non-significant.

a *p* = 0.0125, one-way ANOVA post hoc Tukey’s multiple comparisons test compared to FGF14-GFP DMSO.

b *p* = 0.0009, one-way ANOVA post hoc Tukey’s multiple comparisons test compared to FGF14-GFP DMSO.

c *p* = 0.0002, one-way ANOVA post hoc Tukey’s multiple comparisons test compared to FGF14-GFP DMSO.

**FIGURE 6:**
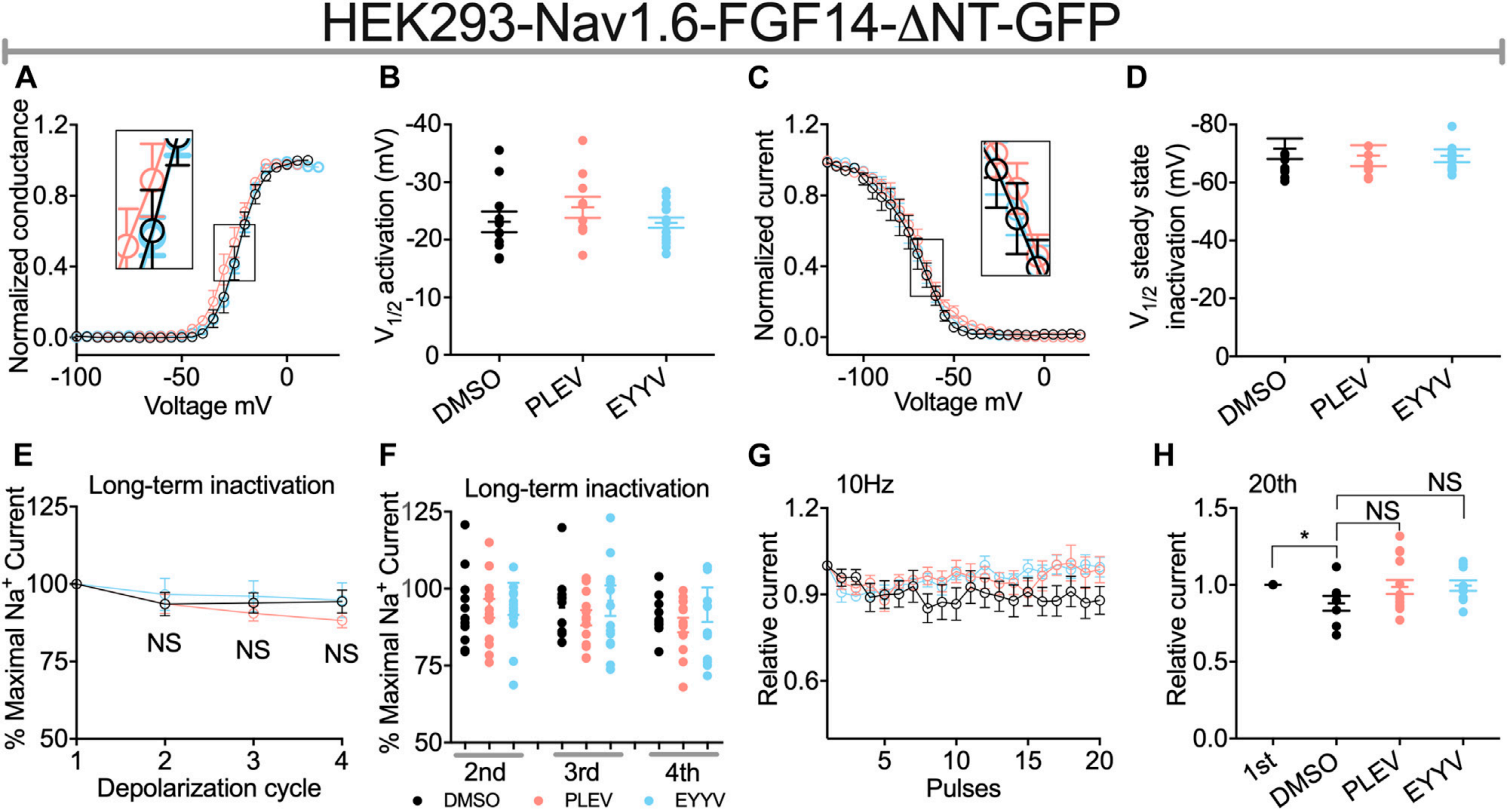
The N-terminal domain of FGF14 is required for peptides to exert modulatory effects on Nav1.6 channel inactivation. **(A)** Conductance-voltage relationships for HEK293-Nav1.6 cells co-expressing FGF14-ΔNT-GFP treated with 0.1% DMSO, 50 µM PLEV and 50 µM EYYV. **(B)** Comparison of V1/2 of activation between the indicated experimental groups. **(C)** Normalized current as a function of membrane potential to characterize the effects of 0.1% DMSO, 50 µM PLEV and 50 µM EYYV on steady-state inactivation. **(D)** Comparison of V1/2 of steady-state inactivation between the indicated experimental groups. **(E)** Ratio maximal INa plotted as a function of depolarization cycle to characterize entry of Nav1.6 channels exposed to 0.1% DMSO, 50 µM PLEV, or 50 µM EYYV in the presence of FGF14-ΔNT. Cells were subjected to four 0 mV 16 ms depolarizations separated by −90 mV 40 ms recovery intervals. **(F)** Bar graph represented 2nd, 3rd, and 4th pulse of LTI. **(G,H)** Assessment of cumulative inactivation of Nav1.6 channels exposed to 0.1% DMSO, 50 µM PLEV, or 50 µM EYYV in the presence of FGF14-ΔNT. It was examined by applying a 2 ms test pulse to −10 mV 20 times at frequency10 Hz from a holding potential of −80 mV. Data are mean ± SEM. Significance was assessed using an Student’s *t*-test comparing cells treated with 0.1% DMSO, 50 µM PLEV and 50 µM EYYV. NS = non-significant; *, *p* at least < 0.05 (detailed *p* values and summary of results are reported in Tables 2, 4).

Similar to their lack of effects on Nav1.6 channel activation in the presence of FGF14, PLEV and EYYV displayed no effects on this electrophysiological parameter in the presence of FGF14-ΔNT (Figures 6A,B). Notably, however, whereas both PLEV and EYYV reversed FGF14-mediated regulatory effects on Nav1.6 channel steady-state inactivation (Figures 4C,D), neither peptide displayed effects on this parameter in the presence of FGF14-ΔNT (Figures 6C,D). Furthermore, the additional modulatory effects of PLEV on LTI and cumulative inactivation observed in the presence of FGF14 were also not observed in the presence of FGF14-ΔNT (Figures 6E–H). Overall, these studies provide strong evidence that these two peptides, despite being derived from the core domain of FGF14, exert their effects on Nav1.6 channel activity through a mechanism dependent upon the presence of the N-terminal domain of FGF14.

**TABLE 4:** Nav1.6 channel LTI in the presence of FGF14-ΔNT and tetrapeptides PLEV, EYYV.

Condition	LTI (% Maximal Na^+^ current)		
	2^nd^ Pulse	3^rd^ Pulse	4^th^ Pulse
FGF14-ΔNT-GFP DMSO	92.82 ± 4.57 (9)	93.21 ± 3.97 (9)	94.17 ± 4.52 (9)
FGF14-ΔNT-GFP PLEV	91.27 ± 3.10 (12)	88.4 ± 2.33 (12)	86.91 ± 2.58 (12)
FGF14-ΔNT-GFP EYYV	98.12 ± 7.34 (10)	97.66 ± 6.87 (10)	93.37 ± 8.12 (10)

Data are mean ± SEM; ns = nonsignificant.

## Discussion

PLEV and EYYV represent previously identified tetrapeptides that correspond to clusters of amino acids on the β12 sheet and β8-β9 loop of FGF14, respectively, that are at the FGF14’s PPI interface with the CTD of Nav1.6 (Ali et al., 2014, 2016; Singh et al., 2020). Given the primacy of these structural motifs of FGF14 in enabling FGF14:Nav1.6 complex assembly, we investigated if short peptides corresponding to these motifs of FGF14 could confer functionally relevant modulation of the Nav1.6 channel macromolecular complex. Given that perturbation of the PPI between FGF14 and the CTD of Nav1.6 gives rise to neural circuitry aberrations that are linked to neurologic and neuropsychiatric disorders (Di Re et al., 2017; Paucar et al., 2020), such peptides could represent promising “small-molecular inhibitor starting points (SMISP)” to develop PPI-targeting neuromodulators (Koes and Camacho, 2012a; 2012b). To pharmacologically evaluate the PLEV and EYYV tetrapeptides as potential SMISPs, we employed an amalgam of complementary and orthogonal approaches including *in silico* molecular modeling, the LCA, and whole-cell patch-clamp electrophysiology. These results demonstrated that PLEV and EYYV both displayed functional modulation of the Nav1.6 channel macromolecular. Despite displaying mostly convergent modulatory effects on Nav1.6 channel activity, PLEV and EYYV displayed some divergent effects, consistent with their derivation from different structural motifs of FGF14. Overall, these studies provide strong evidence that PLEV and EYYV could serve as promising scaffolds for the development of chemical probes targeting the PPI interface between FGF14 and the CTD of Nav1.6. Additionally, this study further support the notion that short peptides derived from “hot spot” (London et al., 2010, 2013) of PPI interfaces could serve as innovative probes to guide drug discovery efforts.

### PLEV and EYYV Disrupt FGF14:Nav1.6 Complex Assembly Through Predicted Interactions With Resides at the PPI Interface

In our previous study (Singh et al., 2020), we pharmacologically evaluated all three tetrapeptides at a single concentration of 50 µM using the LCA. In the FGF14:Nav1.6 wild type condition, all three tetrapeptides displayed comparable single concentration activity. In conditions in which putative “hot spot” residues at the FGF14:Nav1.6 PPI interface were mutated, such as Y158 and V160 (Ali et al., 2016, 2018), the three peptides displayed some divergent effects. For example, in the FGF14^V160A^ condition, all three peptides lost activity, whereas in the FGF14^Y158A^ condition, FLPK retained its inhibitory effects on FGF14:Nav1.6 complex assembly, whereas PLEV and EYYV were shown to increase FGF14:Nav1.6 complex assembly.

Based upon these divergent effects of PLEV and EYYV compared to FLPK, we elected to further pharmacologically evaluate these two tetrapeptides in the present investigation. In dose-response analyses studies, PLEV and EYYV were shown to inhibit FGF14:Nav1.6 complex assembly with IC_50_ values of 41.1 ± 4.4 µM and 35.7 ± 4.7 µM, respectively. These in-cell studies, considered collectively with the *in silico* molecular modeling studies, highlight potential residues of FGF14 that, when occupied by a ligand, inhibit FGF14:Nav1.6 complex assembly. For example, PLEV and EYYV have predicted interactions with residues of the core domain of FGF14 including R117 and E152, as well as predicted interactions with residues of the N-terminal domain of FGF14 including K74. Given these predicted modes of binding coupled with the inhibitory effects of PLEV and EYYV on FGF14:Nav1.6 complex assembly, these residues could represent potential “hot spots” for the development of small molecular modulators targeting the FGF14:Nav1.6 PPI interface.

### PLEV and EYYV Modulates Nav1.6 Channel Activity

To test whether PLEV and EYYV affected Nav1.6-mediated currents, we employed whole-cell patch-clamp electrophysiology in heterologous cell systems. We used HEK cells stably expressing human Nav1.6 (HEK-Nav1.6), and transiently transfected with GFP (HEK293-Nav1.6-GFP) or FGF14-GFP (HEK293-Nav1.6-FGF14-GFP) and treated with 50 µM PLEV and EYYV or 0.1% DMSO (Figures 3, 4). Both peptides did not show measurable effect in the absence of FGF14 (Nav1.6-GFP); however, PLEV partially reversed the FGF14-mediated regulatory effects on peak transient I_Na_ density (Figures 3A–C; Table 1). This effect was not observed due to treatment with EYYV, highlighting the divergent mechanisms of action of the two tetrapeptides. Unlike their differential modulation of FGF14-mediated regulatory effects on peak transient *I*_Na_ density, PLEV and EYYV displayed conserved effects in terms of reversing FGF14-mediated regulatory effects on Nav1.6 channel steady-state inactivation (Figures 4C,D; Table 1). Whereas PLEV and EYYV displayed conserved modulatory effects on Nav1.6 channel steady-state inactivation, PLEV also displayed distinct effects on LTI and cumulative inactivation that were not observed due to treatment with EYYV. Specially, PLEV increased the fraction of Nav1.6 channels that entered into LTI in a FGF14-dependent fashion, indicating a complex mechanism of action where treatment with PLEV results in altered function of FGF14. PLEV similarly altered the function of FGF14 as it related to cumulative inactivation, as HEK293-Nav1.6-FGF14-GFP cells treated with PLEV displayed an increased number of available channels after repetitive stimulation, whereas the same cells treated with DMSO displayed no change in the number of available channels before and after repetitive stimulation. All together, these effects demonstrate that PLEV and EYYV display both convergent and divergent effects on Nav1.6 channel activity, which could be attributable to their derivation from different structural motifs of FGF14 and differential interactions with residues at the FGF14:Nav1.6 PPI interface.

### FGF14-1b N-Terminus Domain Required to Modulate Nav1.6 Channel Activity

The N-terminal domain of FGF14-1b, the splice variant of FGF14 studied in the present investigation, is essential for conferring FGF14-mediated regulation of Nav1.6 channel activity, as deletion of the N-terminus of FGF14-1b abolishes the regulatory effects of FGF14 on a myriad of electrophysiological properties of Nav1.6 channels (Laezza et al., 2007; Yan et al., 2014). In line with previous studies, FGF14-ΔNT potentiates Nav1.6 current densities (Figures 3A–C, 5A–C; Tables 1, 2), causes a depolarizing shift in the voltage-dependence of Nav1.6 channel activation, a hyperpolarizing shift in the voltage-dependence of Nav1.6 channel steady-state inactivation, while having no effects on LTI or cumulative inactivation of Nav1.6 channels (Figures 3, 5E–H; Tables 1, 2) (Laezza et al., 2007, 2009; Ali et al., 2018; Singh et al., 2020). In contrast to the modulatory effects of PLEV and EYYV on Nav1.6 channel activity in the presence of FGF14, the peptides display no effects in the presence of FGF14-ΔNT. As such, while the peptides are derived from clusters of amino acids constituent to the core domain of FGF14, their mechanisms of action are nevertheless dependent upon the N-terminal domain of FGF14. Given the central role of the N-terminus of FGF14-1b in the generation of resurgent I_Na_ and the repetitive firing of action potentials (Yan et al., 2014), these results have important implications for anticipating the functional effects of both tetrapeptides in the native system. As FGF14-1b is highly enriched in clinically relevant brain regions, including the nucleus accumbens (Ali et al., 2018) and hippocampus (Hsu et al., 2016), along with Nav1.6, these peptides that disrupt their complex assembly could represent promising scaffolds for their development of PPI-targeting neuromodulators (Dvorak et al., 2021).

## Conclusion

We have studied the modulatory effects of the tetrapeptides PLEV and EYYV, which correspond to residues of FGF14 that are at its PPI interface with the CTD of the Nav1.6 channel, on FGF14:Nav1.6 complex assembly and the functional activity of the Nav1.6 channel macromolecular complex. We have shown that both peptides functionally modulate Nav1.6 channel activity in a manner dependent upon the N-terminal domain of FGF14. Whereas both tetrapeptides inhibited FGF14:Nav1.6 complex assembly and reversed the FGF14-mediated depolarizing shift in the voltage-dependence of Nav1.6 channel steady-state inactivation, PLEV exerted additional modulatory effects not observed due to treatment with EYYV. Specially, PLEV increased the fraction of Nav1.6 channels that entered into LTI and modulated the fraction of channels that re-open during repetitive stimulation in a FGF14-depedent manner, indicating a complex mechanism of action where treatment with PLEV results in altered function of FGF14. Consistent with the molecular modeling studies shown in Figure 1, these nonoverlapping modulatory effects on Nav1.6 channel activity could be attributable to the tetrapeptides being derived from different structural motifs of FGF14 and resultantly displaying divergent interactions with residues at the FGF14:Nav1.6 PPI interface.

## Data Availability

The original contributions presented in the study are included in the article/supplementary files, further inquiries can be directed to the corresponding authors.
